# Challenges in global advancement of robotic surgery

**DOI:** 10.2340/17453674.2025.44879

**Published:** 2025-10-27

**Authors:** Birger C FORSBERG, Martin GERDIN WÄRNBERG

**Affiliations:** 1 Department of Global Public Health, Karolinska Institutet, Stockholm; 2 Perioperative Medicine and Intensive care, Karolinska University Hospital, Solna, Sweden

Surgery has been under constant development ever since humans made their first attempt to intervene in the human body to save lives and improve health. The human capacity to perform surgery is advancing every day, even over the years when there may have been a position that we have reached the ultimate skills in operating and supporting the patient. This is well exemplified by the rise and continued advancement of robotic surgery—an idea that was considered science fiction 30–40 years ago.

Evidence is now accumulating that robotic techniques are the way forward for surgeons. As has been highlighted in a recent review, robot systems help surgeons to achieve greater precision and perfect implant positioning, greatly affecting both the outcomes after surgery and prosthesis lifespan [1]. Spitzer et al. further underline this when stating that the principal benefits of robotic-assisted surgery are that it allows physicians to achieve better component placement while decreasing procedural outliers and improving precise restoration of the mechanical axis [2].

However, the access to surgery in general and robotic surgery even more so is extremely unequal. Already in 1980, the then Director General of WHO, Dr Halfdan Mahler, said that “the vast majority of the world’s population has no access whatsoever to skilled surgical care and little is being done to find a solution. I beg of you to give serious consideration to this most serious manifestation of social inequity in health care” [3]. Things have improved since then, but as documented by the global surgery initiative there are still so many people in the world who lack access to even the most elementary surgery [3]. One of the reasons is the lack of qualified surgeons, generally in many countries and in rural areas in even more countries [4].

Could robotic surgery be a faster way forward to expand surgery to those populations deprived of such services? Unfortunately, robotic systems require substantial startup investments, along with continuous maintenance and single-use equipment expenses for each procedure as pointed out by Zimnoch et al. Also, the authors underline that the associated expenses restrict smaller or resource-limited institutions from obtaining these systems [1].

This is an observation very relevant to the article in *Acta Orthopaedica* by Ruangsomboon and colleagues [5]. They report on the cost-effectiveness of robot-assisted vs conventional total knee arthroplasty in Thailand. Somewhat disappointingly, the researchers find that in the context of a middle-income country, such as Thailand, broad adoption of robot-assisted knee surgery is not economically attractive compared with conventional surgery for end-stage knee osteoarthritis patients. The study is a point-of-time observation and clearly the conditions for robotic surgery are likely to change significantly over the coming years. The market for surgical robots is exploding in the Asia-Pacific region, and China and India in particular are driving this development. Surgical robots can be expected to become increasingly available, at lower cost. Also, as more surgeons develop skills in robotic techniques the cost of training surgeons in applying the technique will decrease.

Even so, the article by Ruangsomboon et al. points to the importance of having a critical perspective on the adoption of new technology so that it is introduced with due consideration given to the readiness of the local health systems to integrate the technology [5]. There may well be situations in which actors offer equipment at low or no cost, making it attractive for care providers to accept such offers [6]. This does not change the fundamentals of the costs and effectiveness to society of the utilization of the equipment. As surgical robot manufacturing increases, an eye needs to be kept on the development of robot applications so that actual local needs support the adoption of the technique.
